# Findings from the Peutz-Jeghers Syndrome Registry of Uruguay

**DOI:** 10.1371/journal.pone.0079639

**Published:** 2013-11-19

**Authors:** Asadur Tchekmedyian, Christopher I. Amos, Sherri J. Bale, Dakai Zhu, Stefan Arold, Joaquin Berrueta, Natalie Nabon, Thomas McGarrity

**Affiliations:** 1 Gastroenterology, Pasteur Hospital, Ministry of Public Health, Montevideo, Uruguay; 2 Center for Genomic Medicine, Department of Community and Family Medicine, Geisel School of Medicine, Dartmouth College, Lebanon, New Hampshire, United States of America; 3 GeneDx, Gaithersburg, Maryland, United States of America; 4 Department of Biochemistry and Molecular Biology, Center for Biomolecular Structure and Function, MD Anderson Cancer Center, University of Texas, Houston, Texas, United States of America; 5 Department of Gastroenterology, National School of Medicine, Universidad de la Republica, Montevideo, Uruguay; 6 Division of Gastroenterology and Hepatology, Department of Medicine, Milton S. Hershey Medical Center, Pennsylvania State University, Hershey, Pennsylvania, United States of America; 7 Division of Biological and Environmental Sciences and Engineering, Computational Bioscience Research Center, King Abdullah University of Science and Technology, Thuwal, Saudi Arabia; Gentofte University Hospital, Denmark

## Abstract

**Background:**

Peutz-Jeghers syndrome (PJS) is characterized by intestinal polyposis, mucocutaneous pigmentation and an increased cancer risk, usually caused by mutations of the STK11 gene. This study collected epidemiological, clinical and genetic data from all Uruguayan PJS patients.

**Methods:**

Clinical data were obtained from public and private medical centers and updated annually. Sequencing of the STK11 gene in one member of each family was performed.

**Results and discussion:**

25 cases in 11 unrelated families were registered (15 males, 10 females). The average age of diagnosis and death was 18 and 41 years respectively. All patients had characteristic PJS pigmentation and gastrointestinal polyps. 72% required urgent surgery due to intestinal obstruction. 3 families had multiple cases of seizure disorder, representing 20% of cases. 28% developed cancer and two patients had more than one cancer. An STK11 mutation was found in 8 of the 9 families analyzed. A unique M136K missense mutation was noted in one family. Comparing annual live births and PJS birth records from 1970 to 2009 yielded an incidence of 1 in 155,000.

**Conclusion:**

The Uruguayan Registry for Peutz-Jeghers patients showed a high chance of emergent surgery, epilepsy, cancer and shortened life expectancy. The M136K missense mutation is a newly reported STK 11 mutation.

## Introduction

Peutz-Jeghers syndrome (PJS) is a rare hereditary disorder characterized by intestinal polyposis, mucocutaneous pigmentation and increased risk for cancer. The *sine qua non* of the diagnosis of Peutz-Jeghers syndrome (PJS) is the hamartomatous gastrointestinal polyp characterized histopathologically by the unique finding of mucosa with interdigitating smooth muscle bundles in a characteristic branching tree appearance [1]. Cutaneous hyperpigmented macules are rarely present at birth; they become pronounced in most individuals before the fifth year, but then may fade in puberty and adulthood. Histologically, increased melanocytes are observed at the epidermal-dermal junction, with increased melanin in the basal cells. A 93% cumulative lifetime risk of cancer was observed in a large collected series from PJS registries [2] but another series reported a risk for noncutaneous malignancy of 53% [3]. Linkage studies mapped the genetic basis for disease to 19p13.3 [4] and germ line mutations in the serine threonine kinase 11 (STK11) gene have been identified as the major cause of PJS [5]. However, linkage studies suggest that a minority of families with PJS do not have STK11 mutations [6]. Uruguay is a South American country with 3 million habitants, mostly of European origin. The first PJS patient in Uruguay was reported in 1968 [7]. The aim of this study was to collect and analyze epidemiological, clinical and genetic data from all patients with PJS in Uruguay.

## Materials And Methods

All known PJS patients in Uruguay have been included in a National Registry since December 2000. Information about PJS patients was obtained from public and private medical centers or submitted by treating physicians. Patients were contacted personally or through their treating clinicians. Clinical data were input into a digital chart and updated annually. Data was collected from a standardized formal questionnaire developed for this study and a review of clinical records retrospectively [8].

**Figure 1:**
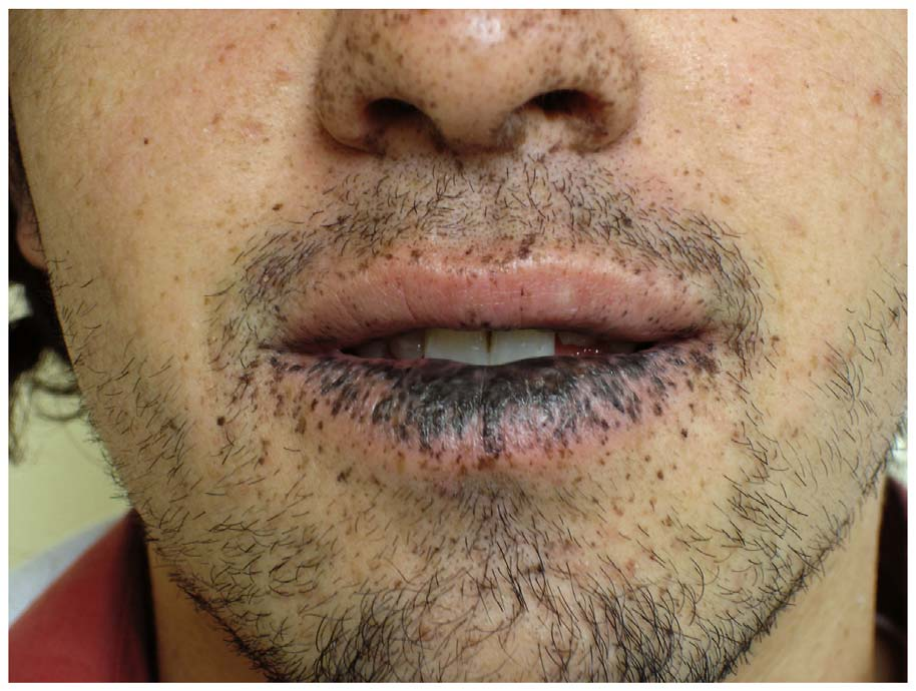
Characteristic peri-oral melanotic pigmentation more pronounced on the lower lip.

The diagnosis of PJS was made according to defined criteria, which include the observation of typical mucocutaneous pigmentation and/or the presence of 2 or more pathognomonic hamartomatous gastrointestinal polyps. In first-degree relatives of clinically diagnosed PJS individuals, observation of mucocutaneous pigmentation was sufficient for diagnosis. This diagnostic criteria follows the working definition of PJS suggested by Giardiello et al. [9] and further refined in a recent consensus meeting [10]. Figure 1 shows representative cutaneous pigmentation in one of our patients. This individual has seen the manuscript and figure and has provided his written informed consent for publication. Endoscopies and polypectomies were done as a result of surveillance in patients with a known family history of PJS or the result of diagnostic colonoscopy due to symptoms.

In a single individual, a clinical diagnosis of PJS may be made when any one of the following is present:

- Two or more histologically confirmed PJ polyps.
- Any number of PJ polyps detected in one individual who has a family history of PJS in close relative (s).
- Characteristic mucocutaneous pigmentation in an individual who has a family history of PJS in close relative (s).
- Any number of PJ polyps in an individual who also has characteristic mucocutaneous pigmentation.

A calculation of PJS incidence was performed using live birth records from 1915 to 2009 with the recorded birth of each case of PJS. The incidence of PJS was also calculated for births before and after 1970.

The study protocol and consent were approved by the ethics committee of the National Medical School at Montevideo, Uruguay and written informed consent was obtained from all patients who underwent genetic analysis. Information on medical history and family medical history was obtained by a standardized 6 page questionnaire. Informed and written consent was obtained from the next of kin on the behalf of 3 minors who participated in the study. The ages of the minors were 12, 14, and 16 years of age.

Peripheral blood samples were collected from one affected member of each available family. Personal details were noted in a questionnaire at the time of blood sample collection. Samples were stripped of individual identifiers and relabeled with a unique random identifying number for subsequent transmission to the laboratory. DNA was extracted from the submitted samples using standard procedures. The genetic analysis was performed in a CLIA-certified laboratory (GeneDx, Gaithersburg, Maryland).

Sequencing of the STK11 gene was performed to investigate the presence of gene mutation. Exons 1–9 of the STK11 gene were PCR-amplified from genomic DNA obtained from the submitted specimen. Bi-directional sequence was obtained and DNA sequence was analyzed and compared to the published gene. Array-based comparative genomic hybridization (aCGH) using a custom designed oligonucleotide array (ExonArrayDx v.1.0) was also performed. The array contains DNA oligonucleotide probes that cover all exons and introns of the STK11 gene. Hybridization data were analyzed with Agilent Technologies software (DNA Analytics) to evaluate copy number at the exon level. The ExonArray is designed to detect most single-exon deletions and duplications. Probe sequences and locations are from the human genome build 18. Positive results were confirmed by quantitative PCR of the STK11 gene. Confirmation of the presence of observed sequence variant (s) was achieved by re-sequencing the identified specific region of the gene using another aliquot of the prepared DNA, to confirm its presence. This was done by either sequence analysis of the specific exon, designed a restriction analyses, or used a quantitative PCR analysis such as MLPA.

## Results

### Clinical Presentation

Twenty-five cases in eleven unrelated families were registered including 15 males and 10 females (Table 1). The age range of participants was between 12 and 65 years of age. Of the index cases, 5 of the 11 families had family history of PJS; in 4 there was no evidence of family history and in 2 the family history was unknown prior to the initiation of this study. The average age at time of diagnosis was 18.3 years (±3.1; minimum  = 2, maximum  = 65). Age at diagnoses differed between sexes, but not significantly (14.6±2.5 for males and 23.8±6.8 for females, p = 0.156). The male to female ratio was 1:0.67.

**Table 1:** Clinical Characteristics and STK 11 Mutation Status.

Family	Gender	Age at Diagnosis	Age at Death/Cause of Death	Age of Cancer Diagnosis/ Cancer Site	Clinical Presentation	Epilepsy	STK 11 Mutation	Site of PIgmentation	Polyp Location- Stomach	Polyp Location- Duodenum	Polyp Location- Small Bowel	Polyp Location- Colon	Family History of PJS
1	Male	7	−	−	Curious pigmentation and polyp	No	(+) deletion one copy	Mouth, eyes, toes, torso	Yes	Yes	Yes	Yes	Yes
	Female	36	44, Metastasis Breast Cancer	33-Thyroid, 36-Breast	Curious pigmentation and gastrointestinal symptoms	No		Mouth, eyes, toe, arm	Unknown	Unknown	Unknown	Yes	No
2	Male	31	−	35-Rectum	Obstruction	No	n/a	Lips	Unknown	Unknown	Yes	Yes	No
3	Female	12	−	−	Obstruction	No	(+) deletion ex. 1	Lips, mouth, hands	Yes	Unknown	Unknown	Yes	Unknown
4	Male	5	−	−	Obstruction	No	(+) deletion ex. 1	Mouth, inferior lip, hands	No	Yes	Yes	Yes	Yes
	Female	2	−	−	Curious pigmentation	Yes		Face, hands, feet	No	No	Yes	Yes	Yes
	Male	10	−	−	Obstruction	No		Lips, eyes, hands, genitals	Yes	Yes	Unknown	Unknown	Yes
5	Male	24	35, Complications of Abdominal Surgery	−	Obstruction	No	n/a	Inferior lip	Yes	Yes	Yes	Unknown	Unknown
6	Male	15	−	−	Obstruction	No	(+) deletion ex. 2–3	Mouth, eyes, toes	Yes	Yes	Yes	Yes	No
7	Male	22	−	−	Obstruction	No	(+) deletion ex. 5	Inferior lip, hands	Yes	No	Yes	Yes	Yes
8	Female	13	−	−	Curious pigmentation	Yes	(+) ivs2+1a>g	Inferior lip	Yes	Yes	Yes	Yes	No
9	Female	54	54, Breast Cancer	54-Breast	Pigmentation, polyposis in all family members who underwent endoscopy	No		Inferior lip	Unknown	Unknown	Unknown	Unknown	Unknown
	Female	26	48, Breast Cancer	40-Breast		No		Lips	Yes	Yes	Yes	No	Yes
	Male	3	27, Meningitis	−		Yes		Inferior lip	No	No	Yes	Yes	Yes
	Male	24	50, Small Bowel Adenocarcinoma	48-Small Bowel		Yes		Mouth	No	No	Yes	Yes	Yes
	Female	17	50, Lung Cancer	50-Lung		Yes		Lips, toes	Yes	No	Yes	Yes	Yes
	Male	3	−	−		No		Face, hands, feet	Yes	Unknown	Unknown	Yes	Yes
	Male	3	−	−		No		Inferior lip	Unknown	Unknown	Unknown	Unknown	Yes
	Male	27	−	−		No		Lips, toes, hands, feet	No	No	Yes	No	Yes
	Male	21	23, Car Accident	−		No		Mouth	Unknown	Unknown	Yes	Unknown	Yes
	Female	5	−	−		No		Inferior lip	Unknown	Unknown	Unknown	Unknown	Yes
	Female	8	−	−		No	(+) m136k missense	Hands, lips	Yes	Yes	Yes	Yes	Yes
	Male	10	−	−		No		Lips	Yes	Unknown	Unknown	Yes	Yes
10	Female	65	−	42-Thyroid, 65-Breast, 60-Kidney	Curious pigmentation	No	No mutation found	Inferior lip, hands	Yes	Yes	Unknown	Yes	No
11	Male	14	−	−	Anemia, polyposis	No	(+) deletion ex. 1	Inferior lip	Yes	No	Yes	Yes	No

**Age of Living Patients (Year 2009)**.

All 25 patients had characteristic PJS pigmentation and from those who had evaluation (either by endoscopy, radiology, capsule endoscopy or surgery) of the gastrointestinal tract all had polyps (*n* = 22). In 3 cases no evaluation of the gastrointestinal tract was performed but these individuals had classical pigmentation and were first degree relatives of other members with both pigmentation and polyposis.

The 3 cases whose GI tracts were not evaluated belong to the same family. Of 16 cases that had a small bowel evaluation all had polyposis. Colon polyps were present in 17 of 19 patients that received evaluation of the colon by colonoscopy. Families 2, 5, and 10 were comprised of a single individual. Genetic testing was not performed in families 2 and 5 and did not show a deletion in family 10. All 3 individuals had characteristic PJS pigmentation and gastrointestinal hamartomas. In families 2 and 10 the hamartomas were diagnosed by the same university hospital pathologist who specializes in gastrointestinal pathology and polyps in family 5 was confirmed by an experienced pathologist at a public hospital. No central pathology reviewed occurred.

In Case 1 (Table 1) a colonic hamartomatous polyp with adenomatous changes and high grade dysplasia was resected at 18 years of age and no recurrence has been seen in 10 years of follow up.

Intussusception, a serious mechanical complication, was noted in eighteen cases (72%). These cases required urgent surgery for small bowel obstruction secondary to the presence of polyps. Eleven cases required more than one surgery (median 2; minimum  = 2, maximum  = 4). In contrast to other series of PJS patients [11], no intussusceptions of the stomach or colon were noted.

Eleven cases (44%) had iron-deficiency anemia, including 40% of 15 males and 50% of 10 affected females.

Five individuals (20%) in 3 separate families had a seizure disorder confirmed by a neurologist. An epidemiological study in Uruguay reports a prevalence of epilepsy of 1.15%. The difference in reported epilepsy in PJS patients compared with the national average is significant (p<0.0001). [12].

Seven patients (28%) developed cancer and two patients had more than one cancer, with breast (N = 4) being the most frequent site. The others sites were small bowel (1), thyroid (2), rectum (1), lung (1) and kidney (1). The average age at first cancer was 43 years (range 33–54).

Other findings found in this group of patients were: testicular calcifications in one case and in another case, low grade fibrosarcoma of the leg that required surgical excision.

Average age for all-causes of death was 41 years. Cancer was the most frequent cause. The causes of death were: cancer (*n* = 5), postoperative complications after urgent surgery due to polyposis (*n* = 1), meningitis (*n* = 1) and car accident (*n* = 1). If the patient that died in the car accident is not included, the average age of death would be 44 years. For cancer patients the average age for death was 49 years (range 44–54). The median time to cancer diagnosis calculated using Kaplan-Meier techniques was 45 years of age and the median time to death was 49 years of age.

### Incidence of PJS in Uruguay

In all, we observed 25 cases of PJS born since 1915 versus 4.7 million live births in Uruguay, 1915–2009, an incidence of 1 in 190,000 live births. This does not account for deaths before entering the registry. Restricting to the period from 1970 to present (14 cases of PJS), during which period minimal loss due to death is expected, yielded an incidence of 6.5 per million births or about 1 in 155,000 live births.

### STK11 Mutation Analysis

**Figure 2:**
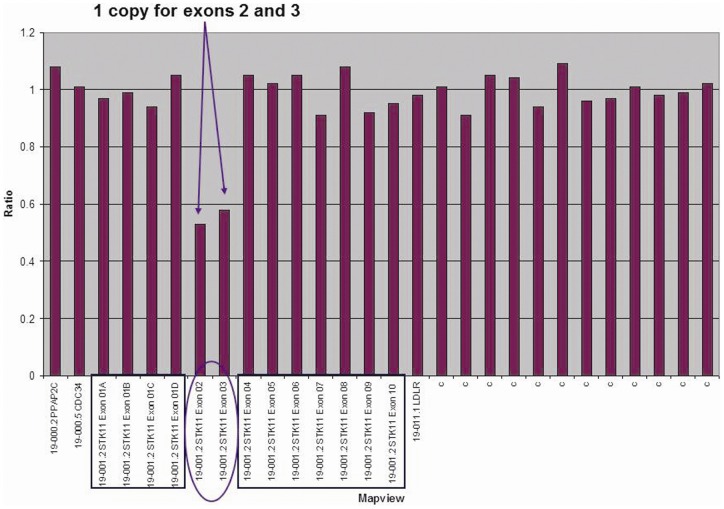
MLPA data shows small intragenic deletion in STK11 gene for patient # 3.

**Figure 3:**
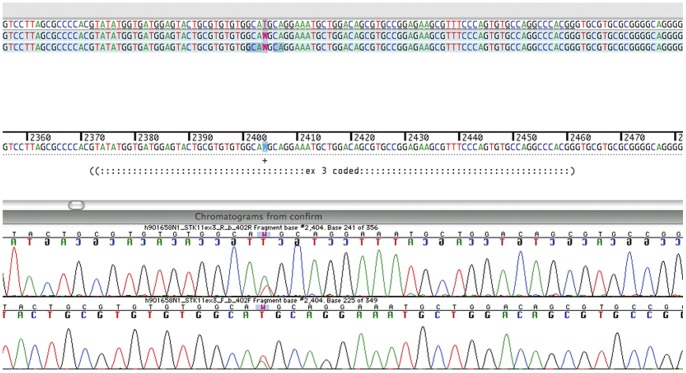
Sequence data and chromatographs for the mutation M136K from patient # 22.

**Figure 4:**
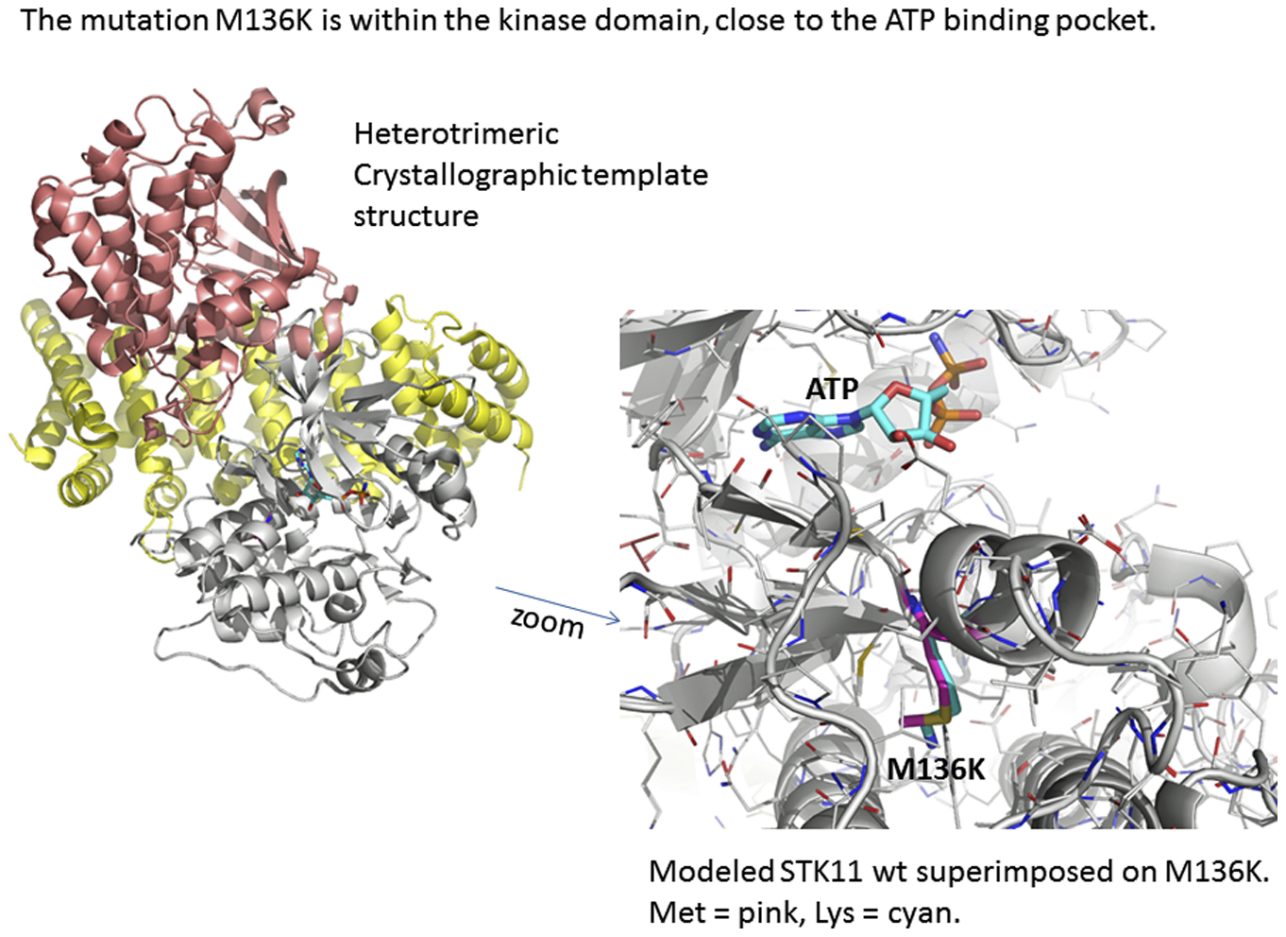
A schematic of the structure of STK11 and novel M136K mutation detected in family 9, patient 22.

A mutation in the STK11 gene was found in 8 of the 9 families analyzed (Table 1). Deletion of all or part of the STK11 gene was identified in 5 patients from 5 families. Targeted array CGH showed that Family 1 had a complete deletion of STK11 while Families 4, 7 and 8 showed deletion of exon 1 and Family 3 had deletion of exons 2 and 3 (results from Multiplex Ligation Probe Amplification is shown in fig. 2). Missense mutations were found in 3 families who did not have deletions. Case 22 was heterozygous for a T>A nucleotide substitution in exon 3, resulting in the replacement of a Methionine codon (ATG) with a Lysine codon (AAG) at amino acid position 136. The sequence data and chromatographs for the mutation M136K (Family 9) are shown on Fig. 3. A schematic detailing the structure of the STK 11 gene and the location of the novel mutation is shown in figure 4. Family 9 had 12 affected family members, 3 of whom suffered from epilepsy (Table 1). Family 8 is heterozygous for an A>G nucleotide substitution at the +1 position of intron 2 of the STK11 gene. Case 9 showed deletion of a Cytosine at position 666 (CGCC{C}GAGA) which results in a frameshift mutation p. Glu223ArgfsX64. Case 24 did not show any detectable mutations either by targeted array CGH or by capillary sequencing of exons 1–9. This case had typical cutaneous pigmentation and confirmed gastrointestinal hamartomatous polyps. She had a history of three different primary cancers (breast, thyroid, kidney) but no family history of PJS.

## Discussion

Peutz-Jeghers syndrome is a rare genetic disorder with an estimated incidence of 1 in 120,000 live births [13]. The lack of national PJS registries makes the estimation of PJS incidence imprecise. Our study of annual and PJS birth rates estimated an incidence of 1/155,000 live births since 1970 and 1/190,000 since 1915, which is in agreement with other reported figures. In this study, we report data from the Uruguayan Registry for PJS patients, focusing on clinical and genetics findings. Uruguay is a country with 3 million habitants, with few tertiary centers. These characteristics allow us to contact each center and to hypothesize that all PJS patients in the country were reached by our registry. We cannot exclude the possibility that some individuals in remote areas of the country may have been missed. In other PJS series the diagnosis of PJS has been made in individuals who lack characteristic mucocutaneous pigmentation. In our series 100% of PJS patients had characteristic pigmentation. Therefore our calculation of incidence may be an underestimation of the prevalence of PJS. Currently, there are 17 individuals with PJS alive and being followed actively. For 16 of these individuals annual follow up has been completed, so that we have data of the ages of onset and phenotypes associated with PJS. Another weakness of our study is that there was no central pathology review conducted.

In familial cases, PJS is inherited in an autosomal dominant manner. The male to female ratio in our study was 1:0.67, which is similar to the ratio reported in Familial Polyposis Coli (1:0.61) [10]. About 50% of probands have an affected parent and about 50% have no family history of PJS [14]. In a Korean series of PJS patients, Choi et al. reported a family history in 47%, no family history in 50% and unknown in 3% [15]. In the current study, 46% of index cases had a positive family history.

Melanotic pigmented macules varying from 1 to 5 mm in size may be found on the vermilion border of the lips, labial mucosa, palate, and tongue [16]. The lips are the most frequent location accounting for the 94.1% of a large series from Japan [10]. In our series, 100% of cases presented with pigmentation of the inferior lip. Hamartomatous gastrointestinal polyps are the hallmark of this syndrome. PJS hamartomatous polyps are characterized histopathologically by the unique finding of mucosa with interdigitating smooth muscle bundles in a characteristic branching tree appearance. These polyps are most prevalent in the small intestine [17], as we described in our patients. In a large series of 182 cases reported from the Mayo Clinic, 96% of patients had small bowel polyps, 60% had colorectal polyps and 24% stomach polyps [18].

Individuals with PJS are at increased risk for intestinal and extraintestinal malignancies. Boardman et al. [3] found that individuals with PJS had a 9.9-fold increased relative risk for cancer; relative risks (RR) were highest for gastrointestinal cancer (RR = 151) and breast cancer (RR = 20.3). Choi et al. [15] reported a mean age at cancer diagnosis in probands of 36 years. In our series the median age at first cancer was later but still young for cancer (43 years) and similar to a recent Dutch study [19], which found the median age at cancer onset to be 45 years of age. The risk for pancreatic cancer is greatly increased over the population risk [8], but there were no reports of pancreatic cancer in our series. Lim et al. [20] reported that 8% and 32% of women with PJS developed breast cancer by age 40 years and 60 years, respectively. In Lim's study all patients were mutation positive. Thus far we have found 2 patients from 2 families that presented with breast cancer at 36 and 65 years old. The youngest died from metastatic breast cancer 8 years after the diagnosis.

In a recent retrospective review from Mayo Clinic, the overall survival of PJS patients was significantly shorter than the expected survival of an age-and gender-matched reference population with a median age of death of 51 years [21]. In a Japanese series comprising 222 PJS patients, 43% of deaths before age 30 were secondary to GI polyps, whereas 60% of deaths over the age of 30 years were due to malignancy [11]. Within our patients, cancer was the most frequent cause of death and the average age for all-causes of death was 41 years. A low grade fibrosarcoma was also reported in our series. Fibrosarcoma has not previously been reported as a clinical feature of PJS, however, STK11 interacts with TP53 in regulation of apoptosis, and dysregulation of STK11 may have some impact on sarcoma risk, as is noted in patients with inherited p53 mutations.

As other polyposis syndromes, PJS may develop extra-intestinal findings. In our series we found testicular calcifications in one patient. Males with PJS are at risk for development of testicular tumors referred to as large cell calcifying sertoli cell tumors [22]. Gastrointestinal bleeding and bowel obstruction are common non-malignant complications of PJS. In our series 72% of 25 patients suffered intussusceptions requiring emergency surgery. Our experience is quite similar to a recently reported Dutch series where 69% of 110 PJS experienced intussusceptions [23]. Anemia was a frequent finding as in other PJS series. Tan et al. [24] reported a prevalence of 42% of patients with anemia (similar to 42% in our population). Anemia is generally related to bleeding from GI polyps and the patients may present with rectal bleeding or hematemesis [17].

*STK11* is a tumor suppressor serine/threonine-protein kinase [25] that controls the activity of AMP-activated protein kinase (AMPK) family members, thereby playing a role in various processes such as cell metabolism and the vascular endothelial growth factor signaling pathway [26], cell polarity [27], p-53 dependent apoptosis [28] and DNA damage response. STK11 acts by phosphorylating the T-loop of AMPK family proteins, leading to activation of PRKAA1, PRKAA2, BRSK1, BRSK2, MARK1, MARK2, MARK3, MARK4, NUAK1, NUAK2, SIK1, SIK2, SIK3 and SNRK but not MELK. STK11 acts as a key upstream regulator of AMPK by mediating phosphorylation and activation of AMPK catalytic subunits PRKAA1 and PRKAA2: it thereby regulates inhibition of signaling pathways that promote cell growth and proliferation when energy levels are low, glucose homeostasis in liver, activation of autophagy when cells undergo nutrient deprivation, and B-cell differentiation in the germinal center in response to DNA damage. STK11 also phosphorylates non-AMPK family proteins such as STRADA and possibly p53/TP53 and acts as a regulator of cellular polarity by remodeling the actin cytoskeleton. STK11 is required for cortical neuronal polarization by mediating phosphorylation and activation of BRSK1 and BRSK2, leading to axon initiation and specification. STK11 is also involved in DNA damage response: it interacts with p53/TP53 and is recruited to the CDKN1A/WAF1 promoter to participate in transcriptional activation. STK11 is able to phosphorylate p53/TP53.

The relevance of such result in vivo is however unclear and phosphorylation may be indirect and mediated by downstream STK11/LKB1 kinase NUAK1 STK1 may also act as a mediator of p53/TP53-dependent apoptosis via interaction with p53/TP53: it translocates to mitochondrion during apoptosis and regulates p53/TP53-dependent apoptosis pathways.

The diverse clinical findings in patients with Peutz-Jeghers syndrome reflects the range of cellular activities that it controls. The most common presenting characteristic of Peutz-Jeghers syndrome relates to the development of hamartomatous polyps. In animal models, haploinsufficiency was found to be sufficient for the development of these polyps. Exact mechanisms that underlie the development of the hamartomas remains unknown, but may relate to overgrowth due to loss in activity for autophagy, or perhaps due to defects in cell polarity, which might cause prolapse of the colonic crypts [29].

The activity of STK11 in neuronal polarization raises the possibility that individuals with STK11 mutations may manifest neurological findings. In addition, individuals with mutations in TSC1, TSC2, and PTEN, all of which influence cAMP activity and the MTOR pathway all display neurological findings and an increased prevalence of epilepsy. In recent papers from Thailand, Korea, Colombia, Hungary and India, epilepsy was not investigated. Moreover a recent systematic review did not mention this clinical association. Despite a lack of previous reporting, 5 of our patients in 3 unrelated families presented with epilepsy (lifetime prevalence of 19%) including 3 of 12 affected family members with the novel M136K missense mutation. Epilepsy was reported in Uruguay by Liard et al. as an association of PJS in 1976 [30] in this family. In a PJS US registry managed by of one of the authors (CA) [8], epilepsy was noted with increased frequency (CA, personal communication). These figures need to be assessed in other series in order to understand its true significance.

Salloch et al. [31] found that persons with truncating mutations had more surgical gastrointestinal surgeries, a higher polyp count, and an earlier age at first polypectomy than persons with non-truncating mutations. In our study, patients 1,3,4,6,7,8 and 9 had truncating mutation. These patients had a median of 1.8 surgeries due to intussusception in contrast to the positive patients with no truncating mutation that had a median of 1 surgery.

A mutation in the STK11 gene was found in 8 of the 9 Uruguayan families analyzed. Deletion of all or part of the STK11 gene was identified in 5 patients from 5 families. We identified a novel mutation, M136K which represents a replacement of a neutral, hydrophobic residue by a charged hydrophilic residue in the serine/threonine protein kinase 11. M136K is likely to destabilize a part of the kinase domain. The part it destabilizes is close to the ATP binding pocket. This may cause the kinase to be less stable, and become more easily degraded, leading to less active molecules. As a result AMPK kinase activation, ATP binding, and hence enzymatic activity by STK11 might be compromised. If so, M136K might act like the G135R mutant, which has been reported as a frequent mutation in melanoma [32], which also compromises control of AMPK. It is also expected that this non-conservative change would impact either protein-protein interactions or the secondary structure of the mutant protein.

In a study of 56 individuals with a clinical diagnosis of PJS in which a combination of sequence analysis to detect point mutations and multiple ligand-dependent probe assay (MLPA) to detect large STK11 deletions was used, STK11 mutation detection rate was 94% [33]. However, the prevalence of germline mutation of the STK11 has been reported with very different frequencies, ranging from 100% [34]–[35] to only10% [36]. STK11 point mutations account for the large majority of PJS cases whereas large deletions account for up to 30% of the cases [37]. STK11 mutations can be found in familial PJS and in some sporadic cases as well [38].

In summary, the analysis of the Uruguayan Registry for Peutz-Jeghers patients showed a high chance of emergent surgery by intestinal obstruction, high prevalence of epilepsy, high risk of cancer and shortened life expectancy. An incidence of 1 in 155,000 live births was recorded in Uruguay. A mutation in the STK11 gene was present in the 89% of families analyzed, and a novel, and previously undescribed mutation, was detected in one.
